# Framing major prebiotic transitions as stages of protocell development: three challenges for origins-of-life research

**DOI:** 10.3762/bjoc.13.135

**Published:** 2017-07-13

**Authors:** Ben Shirt-Ediss, Sara Murillo-Sánchez, Kepa Ruiz-Mirazo

**Affiliations:** 1 Interdisciplinary Computing and Complex BioSystems Group, University of Newcastle, UK; 2 Dept. Logic and Philosophy of Science, University of the Basque Country, Spain; 3 Biofisika Institute (CSIC, UPV-EHU), Spain

**Keywords:** functional integration, origins of life, prebiotic evolution, protocells

## Abstract

Conceiving the process of biogenesis as the evolutionary development of highly dynamic and integrated protocell populations provides the most appropriate framework to address the difficult problem of how prebiotic chemistry bridged the gap to full-fledged living organisms on the early Earth. In this contribution we briefly discuss the implications of taking dynamic, functionally integrated protocell systems (rather than complex reaction networks in bulk solution, sets of artificially evolvable replicating molecules, or even these same replicating molecules encapsulated in passive compartments) as the proper units of prebiotic evolution. We highlight, in particular, how the organisational features of those chemically active and reactive protocells, at different stages of the process, would strongly influence their corresponding evolutionary capacities. As a result of our analysis, we suggest three experimental challenges aimed at constructing protocell systems made of a diversity of functionally coupled components and, thereby, at characterizing more precisely the type of prebiotic evolutionary dynamics that such protocells could engage in.

## Introduction

Living beings on Earth, even in their simplest prokaryote versions, are extremely complex systems, made of a great diversity of molecular components in continuous transformation and interaction. At the base level, each cell is sustained by means of an impressive biopolymer apparatus, which essentially consists of proteins and nucleic acids carrying out complementary tasks to orchestrate an intricate and heterogeneous dynamic organisation with surprising robustness. In addition, this organisation always involves an endogenously synthesized boundary that protects those components/processes from the surrounding milieu and, not less importantly, provides a selective interface that couples them to that external environment. Indeed, all known organisms (genetically-instructed cellular metabolisms) intrinsically depend, both in material and energetic terms, upon a variety of processes that take place across their boundaries – lipid membranes in/on which highly sophisticated mechanisms of transport and energy transduction reside, making possible the maintenance of the system, as a whole, in open, far-from-equilibrium conditions. In a metaphoric sense, a cell is a special type of “nano-factory”, whose molecular machinery conducts chemical syntheses from simpler precursors and uses the products of that complex chemical activity for its continuous reinforcement, managing to re-fabricate the complete synthesis machinery itself.

The problem of origins of life consists in finding a plausible sequence of transitions from abiotic physical and chemical processes towards this level of molecular and organisational complexity, unparalleled by any other phenomena that we observe in the natural world. Therefore, facing this challenge always involves making a strong set of simplifying assumptions, both in terms of the molecular and the organisational features of life as we currently know them. The simplifications tried so far have met with limited success, probably because they represent oversimplifications. From a historical perspective, one can say that the extraordinary success of molecular biology led a whole generation of origin-of-life researchers to believe that the initial steps towards life could be performed by molecules of a single kind (not embedded in a wider chemical organisation). Then, for years, a strong debate was established in the field about, precisely, what kind of molecule (often, what kind of biopolymer) came first, analysing either the abiotic pathway of synthesis that could have brought it about, or the reactive processes that it could have provoked (i.e., the replication or catalytic processes it hypothetically took part in). Fortunately, following the advent of systems biology at the turn of the century, an increasing awareness about the irreducibility of living phenomena to a specific type of molecular mechanism is extending throughout a new generation of scientists, including those interested in the problem of origins [1–2].

In this context, we would like to bring to the fore a fundamental but clearly underappreciated aspect of biological phenomenology: namely, the diversity of components and phase heterogeneity it involves. Aqueous solution chemistry is important for life, but one should not forget that all living organisms require additional physicochemical processes that take place in environments where water is excluded, to different extents. Luckily, we are not alone in the recognition of this basic biological feature: researchers exploring ‘molecular crowding’ [3–5] share the view and criticize, on similar grounds, a significant part of the biochemical knowledge inherited from last century. Membrane biophysicists have also repeatedly complained about the traditional imbalance in biochemistry between the attention given to soluble enzymes over membrane proteins, whose physiological tasks have equal relevance, but are carried out at interfaces or in conditions that are radically different from bulk water (see, e.g., [6]). Even stronger claims about the intrinsic ‘vectorial’ character of metabolism have been made by several authors coming from the field of bioenergetics, who underline the role of chemiosmotic mechanisms for the sustainability of any type of cell [7–8]. Furthermore, this more encompassing approach to life is fully congruent with other insights coming from investigations on reaction–diffusion processes in biology, which have revealed, since the pioneering work of Turing [9], the enormous potential of coupling chemistry with the constrained spatial diffusion of the molecules involved [10–11]. Therefore, given the cellular nature of all life known on our planet, and given the importance of compartmentalized chemistries for understanding many biological phenomena, it may be productive to try origin-of-life simplifications that do not completely erase this aspect at the beginning. The combination of diverse chemical reactions with self-organization and self-assembly processes in heterogeneous, multi-phase conditions could actually be crucial at those first stages: this is the main assumption that most of us working in the ‘protocell camp’ make [12–16].

Under this general hypothesis, one can distinguish two major avenues of research. According to the first, organic compartments of different types (micelles or other colloidal structures) would initially play the role of harbouring surfaces or hydrophobic domains, on which several prebiotic compounds might be adsorbed, in such a way that their chemical reactivity is promoted, leading to more intricate transformation networks and molecular species of various kinds. Several models have been suggested in this direction, from the classical coacervates of Oparin’s [17] and more recent versions of it [18], to the obcell theory of Cavalier Smith’s [19], based on Blobel’s ideas [20], later also revisited by Griffiths [21]. These proposals do not especially favour vesicle compartments, because the encapsulation of the incipient chemistries within a distinct, aqueous micro-environment is not taken to be so relevant at that stage. Quite the contrary, they actually consider that complex biomolecular machinery could be developed outside, to be somehow internalized at subsequent stages [21]. So their main concern is to show how soft hydrophobic clusters and interfaces might have been helpful as aggregating agents, fostering reactions of prebiotic relevance that would be thermodynamically unfeasible in open water solution. In this regard, the former proposals are not very different from other scenarios that have suggested ‘harder’ mineral surfaces as the local settings on which prebiotic chemistry could initially thrive [22–26].

Nevertheless, without denying the important role that all these (hard and soft) surface- or interface-chemistry scenarios could play in order to discover reaction pathways to diverse organic compounds, the majority of ‘compartment-first’ approaches have focused on a second research objective: capturing cell-like behaviours by means of vesicle model systems. Compartmentalization could initially be tried with a two-phase system (e.g., droplets or micro-emulsions) but liposome research techniques, developed during the twentieth century, allowed the in vitro exploration of many – both structural and dynamic – properties of supramolecular assemblies that involve, at least, three-phases (water-membrane-water) and show a striking resemblance to biomembranes, despite their much simpler composition and functional capacities (see [15] for a review). In particular, fatty acid vesicles have become the standard protocell model, not just because of their prebiotic plausibility [27–28], but also because of their remarkable stability as compartments [29–30]; their rapid self-assembly kinetics and amenability to be grown and multiplied under lab conditions [31]; their rich inherent dynamics [32]; and the competition–selection experiments they make possible, if mixed with different liposome populations [33–36]. Thus, the interest of working with these model systems stems from the fact that they provide a very natural connection to real cells, which is attractive both for research groups investigating the chemical roots of biological *organisation* and for others trying to determine the first steps of biological *evolution*.

## Discussion

**Figure 1:**
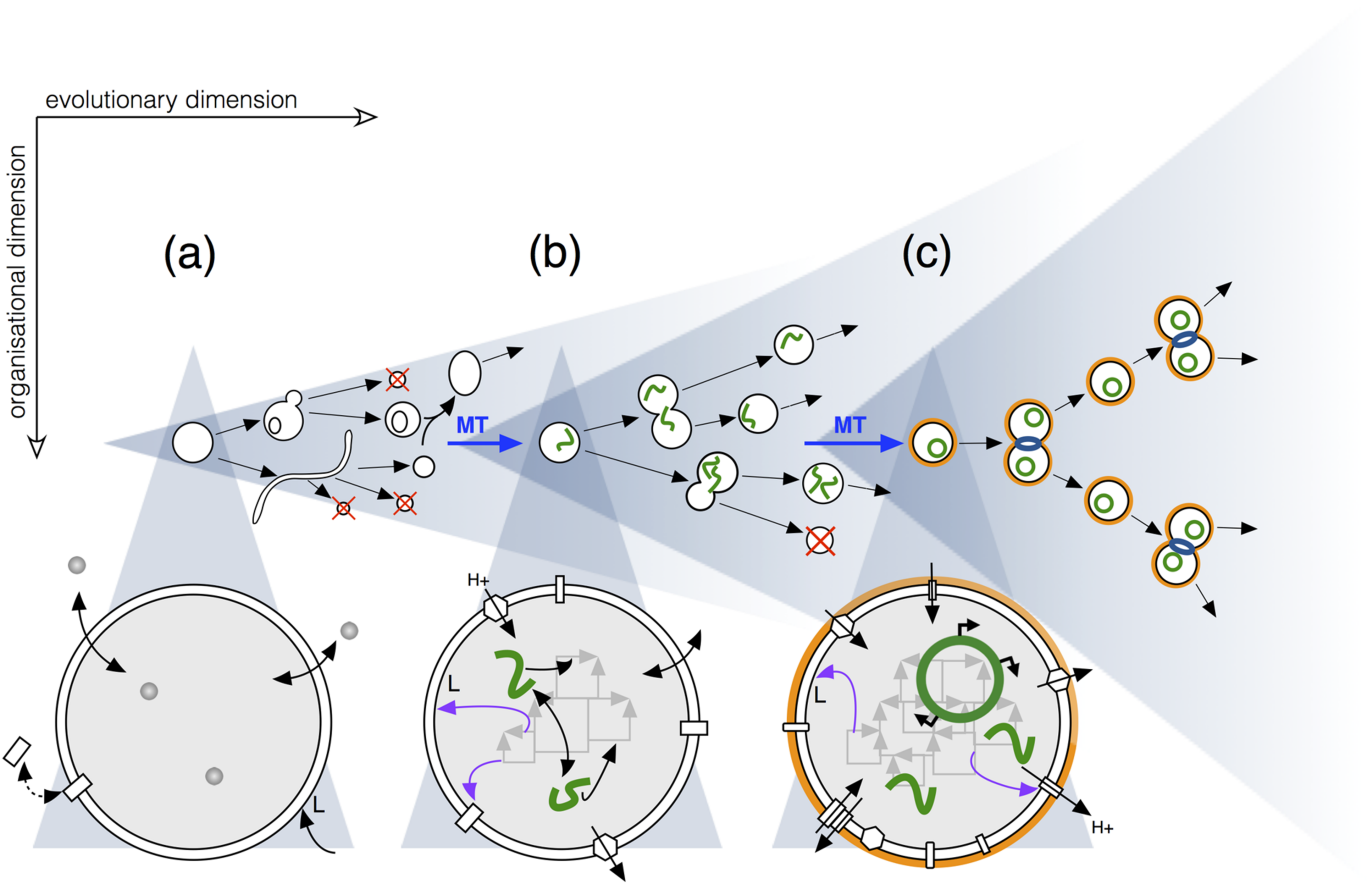
Protocells as the main units of prebiotic evolution: three hypothetical stages of development toward LUCA, with the correlation between protocell organisation and evolutionary potential depicted at each stage. Adapted from [43]. (a) Self-assembled (poly-disperse and likely multilamellar) fatty acid vesicles first start to grow and divide in an unregulated and error-prone way, relying extensively on environmental conditions and external stimuli. (b) After a major prebiotic transition (blue arrow ‘MT’), the first self-producing protocells appeared, able to endogenously synthesise membrane lipids and other membrane components. These protocells, hypothetically making use of the first ‘energy transduction mechanisms’ (leading to precursor ‘energy currencies’ – based on thioesters [62] and/or pH gradients [53], for instance) and a metabolism that incorporated oligonucleotides and oligopeptides (to become RNA and proteins only at a later stage), could activate growth and – still not fully reliable – division cycles more independently of the environment. (c) After a further major prebiotic transition, protocells would reach complexity levels analogous to LUCA’s. Metabolism at that stage already operated on the basis of a ‘genotype–phenotype’ decoupling, with the development of DNA and coding, to enable an open-ended search for new functionalities. The invention of the cell wall and complex protein machinery controlling cell division made reproduction cycles much more coordinated and reliable.

This commentary is aimed at providing a global vision of how these two fundamental aspects of biological phenomenology (the organisational and evolutionary aspects) can be brought together by means of a general scheme of prebiotic transitions that puts ‘protocells’ at the very centre, as the prime axis of the process of biogenesis (see Figure 1). Furthermore, we will defend the view that in order to reconstruct this process a strict ‘bottom-up’ approach should be pursued, starting with chemical precursors of biomolecules, rather than with fully functional biomolecules. Whereas the encapsulation of biopolymers (DNA, RNA, proteins) or cell extracts in self-assembling vesicles of different composition [37–39] constitutes an important proof of principle that biochemistry can be carried out within strongly simplified compartments, these experiments tell us very little about the actual process of origins. A major challenge that must be tackled in order to move the field of origins of life forward would be to couple simple chemistry to prebiotic vesicle dynamics: chemical reactions provide the power for endogenous synthesis and vesicles the adequate scaffolding for the functional integration of what is synthesized. We will proceed briefly with the issue of functional integration below, but the main point to highlight here is that both for reactive processes to become proto-metabolic and for vesicles to become proto-cellular, their mutual, dynamic engagement could well be an early, unavoidable requirement [40].

As Szostak [41] has also noted, the longer we postpone the appearance of chemical encapsulated systems, the more intractable the problem of compartmentalization will surely become. Indeed, if reaction networks could develop their catalytic efficiency in compartment-free scenarios, their eventual encapsulation within lipid vesicles would most probably drive them to self-suffocation, simply because they would run too fast in relation to the (passive) accessibility of nutrients to the internal milieu [42]. The management of osmotic imbalances would be another obvious difficulty, if incipient reaction networks suddenly became incorporated inside semi-permeable membranes [43]. For these reasons, an early ‘co-evolutionary’ scenario in which membranes and internal chemistries develop ‘hand-in-hand’, tightly linked and supportive to each other, makes more sense (see also [44]). Hence our first corollary, expressed in terms of a challenge for the field:

***Challenge 1: coupling chemistry with vesicle dynamics.**** A special effort should be made to discover simple reaction networks whose products include amphiphiles or surfactant molecules that can be spontaneously absorbed by pre-existing vesicles, modifying their basic properties (e.g., their stability, the permeability/fluidity of their membranes) and displacing them, as a result, from their primary quasi-equilibrium states (e.g., inducing their growth and potential reproduction). In turn, vesicle dynamics should prove supportive of – or at least compatible with – that chemistry.*

Cell physiology shows us that endogenous synthesis is a necessary condition to consider a molecular component functional in the most basic biological sense: that is, functional with regard to the (proto-metabolic) organisation that it belongs to. According to this organisational conception, more extensively argued for in [45–46], a component is functional in so far as it contributes in a specific, distinctive way to the overall maintenance of the far-from-equilibrium system that brought it about. Thus, a molecule, taken in isolation, should not be ascribed a function (however, tempted one may be to attribute one to it). Autonomous functionality (orthogonal to the engineering conception of functionality – linked, one way or another, to external human goals) ought to be understood as a relational property to be established and characterized in the context of a dynamic, self-maintaining/self-producing system, in which a diversity of components and processes of interaction come together. In fact, it is most likely that several different types of components/processes were involved in the constitution of the most basic systems with functional parts (in this naturalized, autonomous sense). Determining the minimal number and the specific nature of these prebiotic components/processes (i.e., that ‘irreducible core’ required for functional emergence) remains an open empirical question [46]. One needs to try different combinations of precursors, taking part in various reactive and self-assembling processes, and study their mutual compatibility and overall integration dynamics. We will refer to this as the problem of minimal functional integration in a prebiotic context: namely, the quest to determine the experimental conditions under which the simplest – but at the same time sufficiently robust – systems with autonomous functional components could develop. Arguably, this might be the most urgent question that the field of origins of life should tackle in the near future (also possibly related to what Sutherland [47] calls, in a recent review, the first ‘major system innovation’).

Compartmentalized chemistry, fortunately, is very rich in terms of possibilities for coupling different types of processes and, thereby, its careful exploration is bound to lead us towards proper proto-cellular and proto-metabolic systems (‘a-to-b’ transition in Figure 1). In addition to direct reaction couplings and negative and positive feedback loops (autocatalytic cycles) that can take place within the internal water pool, the presence of closed lipid bilayers strongly restricts the free diffusion of the various soluble species involved, allows the selective passage of precursors and excludes water in limited areas in which an alternative reaction domain is offered (especially for hydrophobic species to interact, or for water-producing reactions to proceed). In recent years, evidence is accumulating to support various potential functions that these self-assembling supramolecular structures could have as reactor promoters and regulators [48–50], i.e., beyond their traditionally ascribed role as selectively permeable enclosures that keep concentrations above critical threshold values. One could mention here, for instance, their catalytic effects on diverse reactive processes (like peptide formation – [51–52]), or the dynamic changes they could provoke in the conditions under which the chemistry takes place (e.g., their capacity to generate pH gradients during growth [53] or the ‘osmotic couplings’ they may induce among internal molecular species via volume changes [54]).

In any case, all these projected or hypothetical functions would only turn real if vesicle compartments effectively contributed to maintain internal chemistries which, in turn, produced a reinforcing effect on the compartments (on their dynamic robustness and/or on their capacity for growth and reproduction). The degree of molecular inter-specificity and functional integration achieved in a first protocellular scenario may be modest, but it is important that both kinetic control and spatial control mechanisms are included in the equation from the beginning, so that they can complement each other in their development. For an interesting bottom-up synthetic-biology example of how this can be approached, see [55].

***Challenge 2: finding conditions and mechanisms for minimal functional integration.**** A focused search for the specific experimental conditions and the set of molecular interaction mechanisms (physicochemical couplings) that lead to minimal functional systems should be pushed forward. The proto-cellular scenario proposed in this commentary makes explicit the need to combine, at least, kinetic and spatial control mechanisms in order to achieve this goal – which would certainly be a major breakthrough, even if the robustness of those initial functional systems proved relatively modest with regard to extant cells.*

Only through time and selection pressure may those initial elementary functions become more refined and intermolecularly specific, leading to stronger modes of functional integration. But in order to walk that pathway, natural selection (NS) and evolutionary dynamics must come to the picture, too. Obviously, it is not legitimate to assume that the exquisite molecular machinery currently responsible for matter transport or energy transduction in cells (for example, ATP-synthases), even if they constitute a common feature across all living domains [7], could be present at the first stages of biogenesis. Such complex membrane mechanisms were, no doubt, latecomers – highly optimized products of evolution. However, any plausible evolutionary explanation of their emergence should begin with simpler lipid compartments and with less efficient, precursor (transport/transduction) mechanisms embedded in them.

Competition–selection experiments carried out among different vesicle populations [33–35] have shown that interactions at that global collective level may be highly relevant from very early stages, long before macromolecular structures, like proteins or nucleic acids, took control of metabolic dynamics. In fact, although the mainstream way to experimentally investigate protocells and their evolutionary capacity has been to take a ‘semi-synthetic’ approach (encapsulating populations of RNA or DNA polymers inside lipid compartments [56–58] or in droplets [59]), we will here propose a more strict ‘bottom-up’ strategy to face this issue, as well. So to speak, everything must come ‘in the same package’: i.e., a deep conceptual shift must also take place to account for the origins of natural selection and proper Darwinian evolution (as explained in more detail in [60]). Instead of using compartmentalization simply as a way to segregate populations of nucleic acids (with the aim to avoid problems like parasitism [61]), the idea here is that integrated protocells constitute the actual units of evolutionary change from the very beginning of the process. Thus, the various stages of vesicle/protocell development should be envisioned in close correlation with differences in the potential for evolution of the populations involved, as schematically shown in Figure 1. In other words, the organisational and evolutionary dimensions of biological phenomena must start unfolding and interweaving very early, feeding on each other, in a scenario where complex biopolymers would be produced by – and incorporated in the workings of – those ‘proto-organisms’ much later. This crudely opens (or re-opens) the question of when should the evolutionary process be called Darwinian (i.e., when NS actually emerges as an operational mechanism), but we consider that the debate ought to take place through an adequate characterization of ‘pre-Darwinian’ competitive/selective dynamics, which remain largely unexplored.

The main advantage of a scheme of transitions like the one portrayed in Figure 1, looking at it from an evolutionary perspective, is that the individuals that lead the process are protocellular systems whose phenotypic space is intrinsically wider than that associated to replicating molecular entities (as in traditional approaches to the origins of life – reviewed in [46] – or in more recent theoretical proposals, like those pointing to the concept of dynamic kinetic stability [63] – see comments below). Protocells constitute ‘scaffolds’ in which a high diversity of functional components may be hooked (including those very replicating molecules but possibly many other simpler ones), leading to multiple state variables and dynamic behaviours for each unit of selection. This endows those systems with the potential to become real Darwinian entities, i.e., organisms (or ‘proto-organisms’, as we called them above) on which natural selection effectively operates [60]. Moreover, major evolutionary bottlenecks in this scenario should not be reduced to a single variable or property but, instead, ought to be related, at least, to the capacity of such systems to: (i) maintain robust dynamics of self-production that underlie their far-from-equilibrium (individual) organization and (ii) reproduce reliably to spread that type of organization in the population. In practice, this entails becoming autonomous from an energetic point of view (hence the importance of setting up the first energy transduction mechanisms [40]) and achieving regularity in the actual process of protocell division, as well as developing mechanisms of heredity (i.e., control of trans-generational variation) [60,64].

A possible – though still tentative – narrative would proceed as follows: initially, (Figure 1a) fatty acid vesicles could self-maintain and grow through the acquisition of external lipid molecules, or by fusing with neighbours, and then divide through a number of pathways, including budding (internal and external) and filamentous intermediates [65]. These growth and division pathways would be largely at the mercy of prevailing environmental conditions and often would lead to a decrease in the mean size of the offspring. Then (Figure 1b) protocells would develop an inner chemistry helping them activate growth and division cycles more independently of environmental factors (first autonomous proto-metabolisms) and avoiding the tendency to decrease in volume at each generation. Nevertheless, such division would be still stochastic, producing a significant amount of non-viable progeny, in a context in which protocell fusion and mixing would still be rife [43]. At later stages (Figure 1c) protocells getting closer to LUCA (the ‘last universal common ancestor’ species) would emerge, with metabolism running now on the basis of more complex (code-mediated) ‘genotype-phenotype’ mappings among functional, subsystem components/modules, all surrounded by an increasingly sophisticated, effective and selective boundary (which would include, at some point, the additional protection of a primitive cell wall). Under these conditions, (i) the space for exploration of new functionalities would widen enormously (getting progressively closer to open-endedness) and (ii) reproduction cycles would become much more reliable, by means of a more elaborate protein machinery specifically devoted to control division processes.

***Challenge 3: characterizing the evolutionary dynamics of pre-Darwinian protocells.**** Rather than focusing on the reaction kinetics and evolutionary dynamics of populations of naked nucleic acid molecules (the core idea underlying the ‘RNA world’ hypothesis), or even compartmentalised chemistries run by poly-nucleotides (e.g., the ‘ribocell’ model), protocell systems with molecular components of much lower molecular complexity should be investigated as units of pre-Darwinian evolution. The overarching question then becomes: how can far-from-equilibrium chemical assemblies that involve low-molecular-weight species be launched in the lab, so that they manage to divide with regularity, explore an ample range of – sufficiently robust – phenotypes, and have potential to set up mechanisms for increasingly reliable heredity?*

It is easy to draw cones, arrows, dead ends, bifurcations and bottlenecks, like we do in Figure 1. Real breakthroughs require the development of experimental strategies and specific protocell models that justify the assumptions and ideas projected through such graphs – or force us to reconsider them. The task is not trivial, though; and not only because the current gap between chemistry and biology is still overwhelming, but also because the devil hides in the details. Prebiotic transitions are particularly tricky due to the fact that the chemical systems involved must work against the natural tendency towards thermodynamic equilibrium (i.e., they must find ways to pay the ‘cost of irreversibility’ as Pascal and colleagues [63] express it). But in order to understand what might be underlying or ‘driving’ those transitions towards higher complexity levels (i.e., the blue arrows signalling the ‘MT’s in Figure 1), one should beware of reductionist or oversimplified explanations. First, as we suggested along the previous lines, a combination of evolutionary and organizational principles should be sought. However, this combination should not be simply conceived on a one-dimensional axis (e.g., in terms of the relative weights of ‘self-organization’ vs. ‘natural selection’ forces, as it has been so commonly done in the past [66–67]). Second, also related to the latter comment, both the actual form of those principles (the main variables and relationships involved) as well as the way they get intertwined should still await the results of ongoing research avenues in the field of systems chemistry [1–2]. For instance, although kinetic control mechanisms must play a central part in the explanation, dynamic kinetic stability [63] is not *the* answer (because replication is not all what matters for evolution, chemical or biological). It is probably too early to draw conclusions and try to make generalizations when we still lack the relevant empirical results (e.g., on the initial set of coupling mechanisms that could transform external sources of free energy into a system’s own means – and sustain, in this way, the first forms of autonomous functionality [46]).

Elucidating the molecular, organisational and evolutionary innovations leading to the major transitions in the process of origins of life will surely require the effort of many research groups in the future. To our eyes, at least, the bottlenecks represented in Figure 1 do not look simple to overcome: we should be aware that the problem is not just developing and coordinating new mechanisms of molecular control, but also implies more complex processes of functional re-organisation and re-integration by the individuals involved, in the context of a constant interaction with other individuals in the population. On these lines, we would like to end this commentary highlighting that ‘protocell population dynamics’, so necessary for the progressive unfolding of phylogenetic (i.e., reliable trans-generational) pathways, are also bound to have other, more immediate proto-ecological implications that could turn very relevant in order to understand those bottleneck transitions. For example, the generation of competitive relationships among different kinds of protocells, could lead to primitive food-webs and diverse modes of selective pressure, and could also be accompanied by other types of symbiotic or collaborative interactions that probably played non-trivial roles in that sense. In fact, those collective dynamics could trigger (through protocell fusion and recombination of complementary components) functional (re-)integration events beyond the minimal compartmentalized chemistries that were under primary focus here. Still regretting Harold Morowitz’s recent passing, we consider that his intuition that «sustained life is a property of an ecological system rather than a single organism or species» [68] should guide future scientific attempts to bring light into the fascinating riddle of biogenesis.
